# DNA Three Way Junction Core Decorated with Amino Acids-Like Residues-Synthesis and Characterization †

**DOI:** 10.3390/molecules21091082

**Published:** 2016-08-23

**Authors:** Claudia Addamiano, Béatrice Gerland, Corinne Payrastre, Jean-Marc Escudier

**Affiliations:** Laboratoire de Synthèse et Physico-Chimie de Molécules d′Intérêt Biologique, UMR CNRS 5068, Université Paul Sabatier, 118 Route de Narbonne, Cedex 9, Toulouse 31062, France; addamiano@chimie.ups-tlse.fr (C.A.); payrastr@chimie.ups-tlse.fr (C.P.)

**Keywords:** three way junction, convertible nucleotide, CuACC, chemically modified oligonucleotides

## Abstract

Construction and physico-chemical behavior of DNA three way junction (3WJ) functionalized by protein-like residues (imidazole, alcohol and carboxylic acid) at unpaired positions at the core is described. One 5′-C(*S*)-propargyl-thymidine nucleotide was specifically incorporated on each strand to react through a post synthetic CuACC reaction with either protected imidazolyl-, hydroxyl- or carboxyl-azide. Structural impacts of 5′-C(*S*)-functionalization were investigated to evaluate how 3WJ flexibility/stability is affected.

## 1. Introduction

The elucidation of the peptide bond formation within ribosome showed that the reaction was catalyzed by ribonucleic acids moieties without any participation of protein amino acids residues in the active site [1,2]. Therefore, it has been tempting to search for nucleic acid catalysts that could perform the reverse reaction, i.e., peptide bond hydrolysis that is commonly assumed by protease under mild conditions. Most of the efforts towards DNA-based artificial protease have been conducted by in vitro selection (SELEX methodology) but to date no unmodified nucleic acid sequences have been successfully selected for that purpose [3]. However, a very recent example from the Silverman′s group showed that DNA featuring protein-like functional groups could be selected for the hydrolysis of an amide bond inserted within an oligonucleotide as substrate [4].

Inspired by the composition of the active site of serine-protease in which three essential amino acids (Histidine, Serine and Aspartic acid) cooperate to achieve the catalytic cleavage of the peptide bond [5], a few groups have developed functionalized nucleosides building blocks bearing imidazole, alcohol and carboxyl groups in order to prepare oligodeoxynucleotides (ODNs) decorated by mimics of the amino acids involved in the triad. Indeed, nucleic acid can be a suitable scaffold for creating a catalytic site made of these appended functionalities since DNA folding into various secondary structures can be easily predicted following the Watson-Crick rules.

With the aim to use in vitro selection methods, the Hollenstein′s group developed triphosphate deoxynucleosides bearing the amino acids functionalities grafted on the purine or pyrimidine base at a position that would interfere neither with polymerase nor with duplex formation ability [6]. It turned out that these modified triphosphates were good substrates for various DNA polymerases and were fully compatible for the selection process [7].

On another hand, the Madder′s group chose to start from 2′-amino-uridine whereby the pendent amine served as a handle for further synthetic functionalization through amide formation with the functional residues properly protected [8]. After conversion into phosphoramidite derivatives adapted to the automated oligonucleotide synthesis, this group built duplexes decorated with imidazole, primary hydroxyl and carboxylic groups within the major groove of the helix [9].

However, to date no artificial protease activities have been reported from these groups. It is clear that selection process with modified triphosphates is a highly difficult task and that duplexes may not offer a sufficient conformational space variability to promote the substrate accommodation and hydrolysis. Here we present our efforts towards the synthesis of a DNA three way junction (3WJ) with three non-pairing thymidines at the core of the structure leading to each strand being specifically modified with an imidazole, hydroxyl or carboxylic pendent functionality at the 5′-C-position of the sugar moiety, respectively. 5′-C-alkyl modifications could be introduced at any position within the ODN and are mostly investigated to enhance nucleic acids analogs stability against nucleases [10]. As the carbon in position 5′ is chiral, we focused our efforts towards the synthesis of the (5′*S*)-5′-C-modified epimer as we showed that this configuration allowed the pendent functionality to lie across the minor groove without altering significantly the Watson and Crick base pairing in duplexes [11]. Moreover, diverging from the concept of using rigid DNA duplex grooves as scaffold where chemical reactions could take place, 3WJ can be applied as a three-dimensional structure that is recognized to be flexible [12,13] and yet stable enough to accommodate modifications. Indeed, the core has been used as a yoctoliter-scale reactor [14] or location to install a metal complex [15]. Finally, the 3WJ with unpaired nucleotides at the core were designed in such a way that the structure becomes flexible enough to build DNA tetrahedrons [16,17].

## 2. Results and Discussion

**Figure 1:**
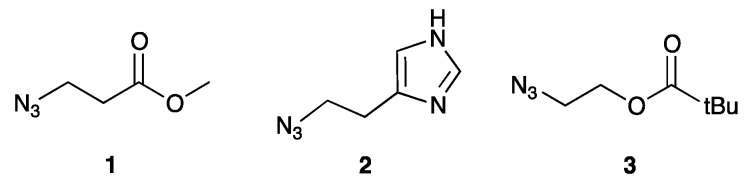
Azides used for introduction of aspartic acid (**1**), histidine (**2**) and serine (**3**) functional residues.

**Scheme 1:**
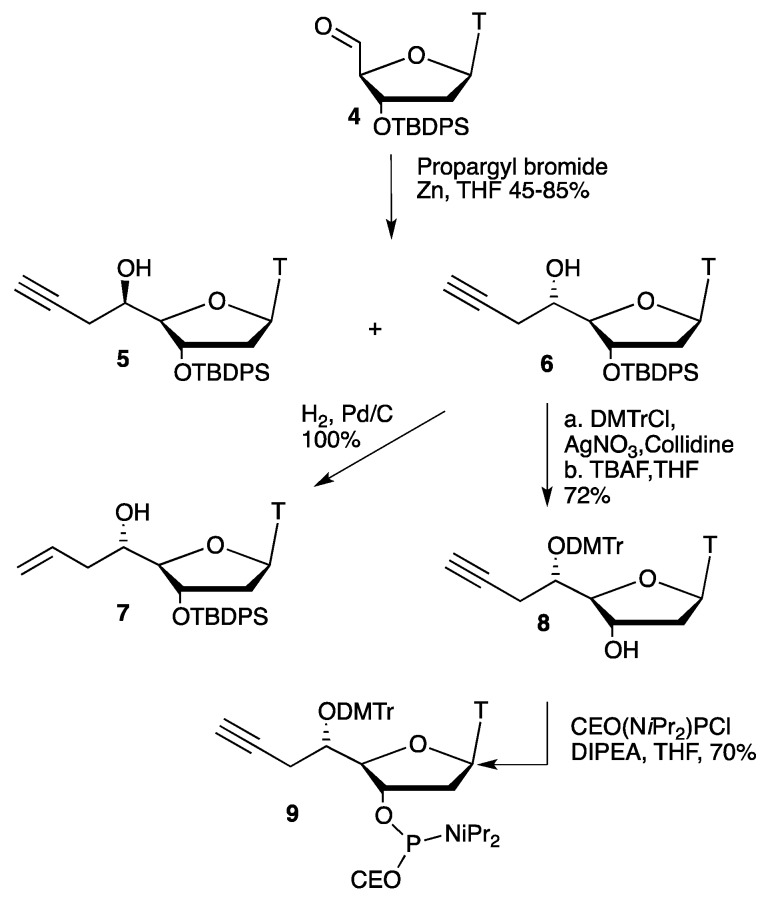
Synthesis of (5′*S*)-5′-C-propargyl-thymidine phosphoramidite **9**.

**Scheme 2:**
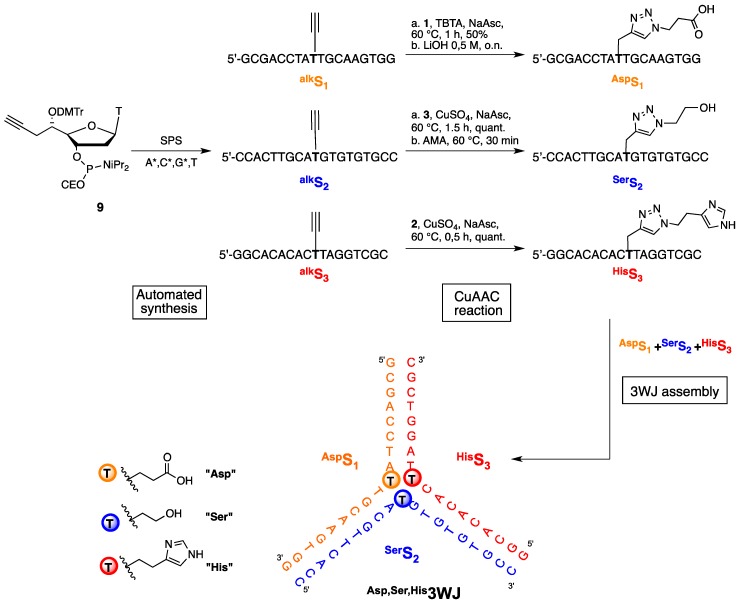
Synthesis of amino acid residues-functionalized oligodeoxynucleotides (ODNs) by SPS with T and protected A*, C*, G* phosphoramidites followed by copper(I)-catalyzed alkyne-azide cycloaddition (CuAAC) reaction and annealing of protein side chains-like functions decorated three way junction **^Asp,Ser,His^3WJ**.

Introduction of functionality within ODNs can be done either by a post synthetic reaction with a convertible nucleotide possessing a reactive group orthogonal to the ODN synthetic process or by synthesis of phosphoramidite building blocks of functionalized nucleosides. However, in the latter case, each different modification requires the synthesis of the corresponding suitably protected nucleotide that must survive the automated DNA synthesis conditions. Therefore, we turned our attention to a simple convertible nucleotide, 5′-C-propargyl thymidine **9** (Scheme 1 and Scheme 2) that can be easily introduced into ODNs by the phosphoramidite technology. A subsequent copper(I)-catalyzed alkyne-azide cycloaddition (CuAAC) [18,19] would then allow the introduction of the desired amino acid like functions since the corresponding azides **1** [20] (for carboxyl) and **2** [21] (for imidazole) have been already described, and **3** (for primary hydroxyl) being readily accessible from commercially available bromoethanol (Figure 1).

### 2.1. Synthesis of Convertible Nucleotide

The synthesis of the key (5′*S*)-5′-C-propargyl-thymidine phosphoramidite **9** started from the known 3′-*O*-*tert*-butyldiphenylsilyl-thymidine aldehyde **4** [22,23] that was reacted under Barbier′s conditions with propargyl bromide to provide a 3/7 diastereoisomeric mixture of (5′*R*)-5′-C and (5′*S*)-5′-C-propargyl-thymidine **5** and **6**, respectively [24,25] (Scheme 1). The separation of the diastereoisomers required a tedious HPLC process to cleanly obtain the fast moving and major compound **6**. The stereochemical assignment of the 5′-C-thymidine nucleoside was done through the conversion of **6** to its corresponding 5′-C-allyl-thymidine **7** by a mild catalytic hydrogenation. The NMR spectra of **7** were identical to those of the previously synthetized 5′-C-allyl-thymidine and assigned to be the (5′*S*)-5′-C-derivative [26]. Subsequent installation of the 4,4′-dimethoxytrityl group on the secondary 5′-hydroxyl function [27] by use of silver nitrate in collidine followed by treatment with fluoride ion provided **8** in 72% overall yield. The free 3′-hydroxyl function of **8** was reacted with 2-cyanoethyl-*N*,*N*-diisopropylchlorophosphoramidite and (5′*S*)-5′-C-propargyl-thymidine phosphoramidite **9** was obtained as a diastereoisomeric mixture (^31^P-NMR δ: 149.7 and 148.8 ppm) in 70% yield after silica gel purification.

### 2.2. Synthesis of Functionalized ODNs

With (5′*S*)-5′-C-propargyl-thymidine phosphoramidite **9** in hands, we engaged the automated ODNs synthesis following the phosphoramidite technology without any change in the protocol used for the corresponding unmodified ODNs (**^wt^S_1_**, **^wt^S_2_** and **^wt^S_3_**) [28]. The (5′*S*)-5′-C-propargyl-thymidine **9** (**T**) was cleanly incorporated in the middle of three different sequences **^alk^S_1_** (5′-GCGACCTA**T**TGCAAGTGG), **^alk^S_2_** (5′-CCACTTGCA**T**GTGTGTGCC) and **^alk^S_3_** (5′-GGCACAC**T**TAGGTCGC), that were purified by means of reverse phase HPLC and characterized by mass spectroscopy in MALDI-TOF mode (**^alk^S_1_** measured 5577.0 [calculated +38.0, found +38.4/**^wt^S_1_**: 5538.6]; **^alk^S_2_** measured 5806.7 [calculated +38.0, found +37.0/**^wt^S_2_**: 5769.7] and **^alk^S_3_** measured 5520.8 [calculated +38.0, found +37.2/**^wt^S_3_**: 5483.6], (Figures S1–S3, Supplementary Materials). The sequences were chosen to be half complementary of each other in order to form a three way junction when mixed together with three non-pairing modified thymidines (**T**) at the core of the structure (Scheme 2 and Table 1).

Arbitrarily, the amino acids functionalities were installed by reacting, in a CuAAC reaction, the (5′*S*)-5′-C-propargyl-thymidine within **^alk^S_1_**, **^alk^S_2_** or **^alk^S_3_** with the azide derivatives **1**, **3** and **2**, respectively, to generate **^Asp^S_1_**, **^Ser^S_2_** and **^His^S_3_**. The CuAAC proceeded cleanly in a quantitative yield between the azides (3 eq.) **3** and **2** and the sequences **^alk^S_2_** and **^alk^S_3_**, respectively, by using a mixture of CuSO_4_ (0.4 eq.) and sodium ascorbate (2 eq.) at 60 °C for 0.5 to 1.5 h [29,30]. **^His^S_3_** was obtained after desalting and **^Ser^S_2_** was recovered after concentrated ammonia/aqueous methylamine (AMA) treatment for pivaloyl protective group removal prior to purification on Sephadex G-25 columns.

Surprisingly, CuAAC reaction of **1** with **^alk^S_1_** failed under the same conditions (whereas it reacted after 3 h with a 70% conversion on **^alk^S_2_** or **^alk^S_3_** as control) and required the use of a commercially available Cu(II)-*tris*(benzyltriazolylmethyl)amine (TBTA) complex to ensure a modest 50% yield of protected **^Asp^S_1_**. The methyl ester was cleaved by a 0.5 M lithium hydroxide treatment overnight and **^Asp^S_1_** was isolated after reverse phase chromatography.

The amino acid residues decorated sequences were characterized by means of mass spectroscopy in MALDI-TOF mode and exhibited the correct mass improvement with respect to their alkyne precursor (**^Asp^S_1_** measured 5692.3 [calculated +115.1, found +115.7/**^alk^S_1_**]; **^Ser^S_2_** measured 5893.7 [calculated + 87.1, found +85.9/**^alk^S_2_**] and **^His^S_3_** measured 5660.4 [calculated +137.1, found +138.8/**^alk^S_3_**]), (see Table 2 and Figures S4–S8 in Supplementary Materials).

### 2.3. Three Way Junctions Assemblies and Thermal Denaturation Studies

**Figure 2:**
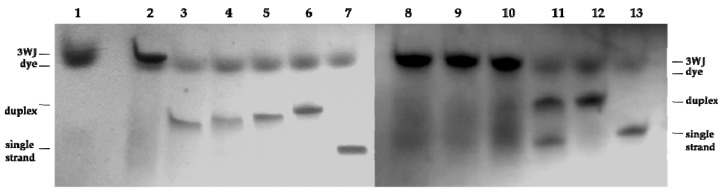
Native PAGE analysis of three way junctions (3WJs). Lane 1: xylene cyanol dye (visible in each lane); lane 2: **^wt^3WJ** (**^wt^S_1_** + **^wt^S_2_** + **^wt^S_3_**); lane 3: **^wt^S_3_** + **^wt^S_1_**; lane 4: **^wt^S_3_** + **^wt^S_2_**; lane 5: **^wt^S_1_** + **^wt^S_2_**; lane 6: **^wt^S_1_** + **^wt^S′_1_**; lane 7: **^wt^S_2_**; lane 8: **^wt^3WJ** (**^wt^S_1_** + **^wt^S_2_** + **^wt^S_3_**); lane 9: **^alk^3WJ** (**^alk^S_1_** + **^alk^S_2_** + **^alk^S_3_**); lane 10: **^Asp,Ser,His^3WJ** (**^Asp^S_1_** + **^Ser^S_2_** + **^His^S_3_**); lane 11: **^alk^S_1_** + **^alk^S_2_**; lane 12: **^alk^S_2_** + **^wt^S′_2_**; lane 13: **^alk^S_1_**.

The duplex and three-way junction formation ability of these modified ODNs were first investigated through polyacrylamide gel electrophoresis analysis (PAGE) in non-denaturing conditions. Mixing equimolecular amount of each strand either for duplex or 3WJ formation showed unambiguously that none of the appended functionality prevent the duplex or 3WJ formation (Figure 2). All of the 3WJs present a single band of identical mobility (lanes 2, 8, 9 and 10) and migrated slower than the fully complementary duplexes (lane 2 vs. lane 6, lanes 8 and 9 vs. lane 12) as expected. The three 3WJs were exclusively formed as the presence of neither half complementary duplexes (lane 2 vs. lane 3, 4 and 5; lane 9 vs. lane 11) nor single strands (lane 2 vs. lane 7, lane 9 vs. line 13) could be detected.

It is noteworthy that all circular dichroism spectra recorded for the modified structures (duplex or 3WJ) were identical to those of their unmodified counterparts.

In order to get insight into the structural impact of the 5′-C(*S*)-functionalization of thymidine within secondary structures, we determined by UV thermal denaturation studies, the melting temperature of duplexes with each modification and three-way junctions with one to three modifications at the core positions, in comparison with unmodified structures denoted as **^wt^S_i_**/**^wt^S′*_i_*** (1 < *i* < 3) for duplexes and **^wt^S_1_**/**^wt^S_2_**/**^wt^S_3_** for **^wt^3WJ**. We then sought to confirm our hypothesis that 3WJ presenting 5′-C-modified unpaired thymidines at its core was a suitable secondary structure scaffold combining both flexibility and stability.

A quick inspection of the B-DNA helical structure indicates that a substituent at the 5′-*C* with the “*S*” configuration will point into the minor groove and therefore would compete with the solvating water molecules and also could interact with opposite phosphate groups depending on the length of the grafted chain. It has been shown that such modifications including alkyl [31], allyl, hydroxypropyl or aminomethyl [32], within oligonucleotides induced significant loss in thermal stability of duplexes built with DNA counterparts. Only aminopropyl-aminopentenyl substituent [11] exhibited a slight improvement in the thermal stability that could be attributed to a favorable interaction of the protonated amino group with the opposite phosphate group. Here, we observed that the propargyl group destabilized the duplex formed with **S_1_**, **S_2_** and **S_3_** in comparison with unmodified duplex by 1 or 2 °C, and that was in accordance with the effect observed for alkyl or allyl substituted thymidine (Table 1). Interestingly, due to their differences in pKa values introduction of the amino acid like functionalities modulate the generally negative effect on duplex melting temperature induced by 5′-C-modifications. Whereas the steric hindrance induced at the 5′-C-position were in all cases increased after cycloaddition compared with the starting propargyl group, the measured melting temperature were not dramatically impacted. With a potential repulsive effect between the carboxylate group and neighboring phosphates, the aspartic residue within **^Asp^S_1_**/**^wt^S′_1_** duplex decreased the melting temperature by 1 °C compared with the propargyl residue and by 2 °C with respect to the unmodified duplex (**^wt^S_1_**/**^wt^S′_1_**). The primary alcohol installed within **^Ser^S_2_** did not modify the behavior of the duplex (**^Ser^S_2_**/**^wt^S′_2_**) compared with its propargyl precursor and remained 1 °C less stable than the natural duplex (**^wt^S_2_**/**^wt^S′_2_**). As previously observed, when positioned towards the duplex major groove [33], the pending imidazole moiety induce a positive effect and compensate the negative one induced by the 5′-C-*(S)*-functionalization, and the overall stability of the duplex made with **^His^S_3_/^wt^S′_3_** was identical to that of the wild type duplex (**^wt^S_3_**/**^wt^S′_3_**) and superior by 2 °C to its propargyl analogue.

The three arms of the designed 3WJ are of identical length and as a consequence we observed for the unmodified **^wt^S_1_**/**^wt^S_2_**/**^wt^S_3_** junction a single and sharp transition at 43 °C. It is noteworthy that in our case, the UV-thermal denaturation study can be used to testify that the 3WJ was formed because when mixing only two strands, either no transition was depicted or there was a tiny transition with a maximum of first order derivative around 31 °C.

In order to investigate the influence of the core functionalization on 3WJ stability, we evaluated different combinations of propargyl or amino acids like residues modified strands with their unmodified counterparts.

All of the combinations made with one or two propargyl-modified strands and unmodified ones to form 3WJ did not change the thermal stability of the junction and exhibited mostly identical *T*m (43 °C). Moreover, the close proximity of the three alkyne groups within **^alk^S_1_**/**^alk^S_2_**/**^alk^S_3_** 3WJ did not provide any additional thermal stability that could have been expected through favorable stacking interaction of the electron rich moieties.

As observed for duplex, the carboxylate function was slightly destabilizing the 3WJ by 1 °C while hydroxyl or imidazole group brought a smooth +1 °C increase of the 3WJ *T*m. Interestingly, the unfavorable impact of the negatively charged carboxylate function was compensated either by the hydroxyl or the imidazole presence and the value of the *T*m of the unmodified 3WJ was recovered unchanged (+43 °C). However, combination of hydroxyl and imidazole was still gently favorable but no additive effect was observed and the *T*m remained close (in the region of +1 °C) to that of the unmodified 3WJ. Eventually, the **^Asp,Ser,His^3WJ** exhibited the same Tm of 43 °C than **^alk^3WJ** and **^wt^3WJ**.

These results suggest that either the core is large enough or/and that the pending groups can not directly interact with each other at least to interfere with the 3WJ stability.

## 3. Experimental Section

### 3.1. General and Nucleosides Synthesis

Reactions were conducted under an atmosphere of argon when anhydrous solvents were used. All solvents were distilled and dried before use. All reagents were obtained from commercial suppliers and were used without further purification. Products were purified by medium pressure liquid chromatography apparatus through 15 µm silica. CDCl_3_ or C_6_D_6_ were used as NMR solvent as well as internal standards for ^13^C- and ^31^P-NMR.

*3′-O-tert-butyldiphenylsilyl-5′-C-propargyl-thymidine* **5** *and* **6**. Zinc powder (0.95 g, 0.015 mol) was activated by sequential addition and removal of HCl 1N, distilled water, ethanol and ether and was suspended together with dissolved compound **4** (2 g, 4.2 mmol) in anhydrous THF (8 mL) under N_2_ atmosphere. Propargyl bromide (1.4 mL, 3.3 mmol (80% toluene solution) was thereafter added dropwise during 30 min at 10 °C. Reaction was monitored by TLC and 20 min after complete addition was quenched by the addition of ethyl acetate (100 mL) and saturated NaHCO_3_ (20 mL). The organic phase was washed with water and brine and dried over MgSO_4_. The solvents were evaporated under vacuum to obtain the crude mixture as a yellow foam (2.17 g; R_f_ = 0.44 ether/petroleum ether (9:1)). The crude product was purified on a silica column and a white foam was obtained after evaporation of solvents (1.33 g, 61% yield). The two diastereomers were separated by preparative silica HPLC (CH_2_Cl_2_/ethyl acetate, 85:15, analyzed on SunFire Silica (5 μm, 4.6 mm × 150 mm)). Five separate injections (about 250 mg each) on a preparative SunFire Silica column (5 μm, 19 mm × 150 mm) were needed to achieve an efficient separation. The two diastereomers **6** and **5** were obtained as white foams in a 70:30 ratio.

*3′-O-tert-Butyldiphenylsilyl-5′-C-(R)-propargyl thymidine* **5**. ^1^H-NMR (300 MHz, CDCl_3_), δ_ppm_: 8.06 (s, 1H, NH); 7.67–7.63 (m, 4H, Ar); 7.47–7.36 (m, 7H, Ar); 7.29 (d, 1H, *J* = 1.1 Hz, H6); 6.24 (dd, 1H, *J* = 8.3, 6.5 Hz, H1′); 4.50 (m, 1H, H3′); 4.01 (dd, 1H, *J* = 3.5, 1.2 Hz, H4′), 3.77 (ddd, 1H, *J* = 7.7, 5.4, 3.5 Hz, H5′), 2.16–2.11 (m, 2H), 2.00–1.93 (m, 3H), 1.86 (d, 3H, *J* = 1.1 Hz, Me7), 1.01 (s, 9H, *t*Bu). ^13^C-NMR (75 MHz, CDCl_3_), δ_ppm_: 163.9; 150.6; 137.3; 136.1; 136.0; 133.3; 133.2; 130.3; 128.12; 128.08; 111.3; 89.5; 87.3; 80.8; 72.9; 71.6; 70.4; 40.1; 27.1; 23.5; 19.3; 12.7. MS-CI (*m*/*z*): 519.1 [M + H]^+^.

*3′-O-tert-Butyldiphenylsilyl-5′-C-(S)-propargyl thymidine* **6**. ^1^H-NMR (300 MHz, CDCl_3_), δ_ppm_: 8.55 (s, 1H, NH), 7.64–7.60 (m, 4H, Ar), 7.47–7.35 (m, 7H, H6, Ar), 6.20 (dd, 1H, *J* = 8.3, 6.1 Hz, H1′), 4.46 (ddd, 1H, *J* = 5.2, 2.4, 2.0 Hz, H3′), 4.00 (t, 1H, *J* = 1.8 Hz, H4′), 3.27 (ddd, 1H, *J* = 7.5, 6.6, 1.7 Hz, H5′), 2.37 (ddd, 1H, *J* = 16.8, 7.5, 2.7 Hz, H6′), 2.26 (ddd, 1H, *J* = 13.3, 8.2, 5.2 Hz, H6′), 2.22 (ddd, 1H, *J* = 16.8, 6.7, 2.5 Hz, H6′′), 2.19 (ddd, 1H, *J* = 13.3, 6.1, 2.7 Hz, H6′′), 1.99 (t, 1H, *J* = 2.6 Hz, H8′), 1.85 (d, 3H, *J* = 1.1 Hz, Me7), 1.07 (s, 9H, *t*Bu). ^13^C-NMR (75 MHz, CDCl_3_), δ_ppm_: 163.8; 150.5; 137.7; 135.9; 135.8; 133.3; 133.1; 130.21; 130.17; 128.0; 111.1; 88.2; 88.0; 80.5; 74.7; 70.7; 69.7; 39.7; 27.0; 24.4; 19.1; 12.5. MS-CI (*m*/*z*): 519.1 [M + H]^+^.

*5′-O-4,4′-Dimethoxytrityl-5′-C-(S)-propargyl-thymidine* **8**. AgNO_3_ (1.12 g, 6.58 mmol, 2.5 eq.) was dried under high vacuum before additions of 3′-*O*-*tert*-butyldiphenylsilyl-5′(*S*)-propargyl-thymidine **6** (1.34 g, 2.6 mmol) and DMTrCl (2.26 g, 6.58 mmol, 2.5 eq.) were made. Collidine (3.19 g, 26 mmol, 10 eq.) was thereafter added under argon atmosphere and the mixture was diluted with freshly distilled THF (50 mL) and left stirring for 17 h. The silver nitrate salts were filtered off and the filtrate was washed with brine (~25 mL), dried on MgSO_4_ and concentrated under reduced pressure. The crude product was thereafter without further purification re-dissolved in freshly distilled THF (20 mL) and a 1 M solution of TBAF in THF (3.15 mL, 3.15 mmol, 1.25 eq.) was added. The mixture was stirred under argon for 16 h and the solvent was evaporated. The crude product was purified by column chromatography (CH_2_Cl_2_/ethyl acetate 8:2, 0.5% Et_3_N to 99.5% ethyl acetate, 0.5% Et_3_N) to yield the pure product as a white foam (1.09 g, 72% yield). ^1^H-NMR (300 MHz, CDCl_3_), δ_ppm_: 7.44–7.21 (m, 10H, H6, DMTr), 6.89–6.77 (m, 4H, DMTr), 6.27 (dd, 1H, *J* = 6.8, 6.3 Hz, H1′), 4.65–4.59 (m, 1H, H3′), 3.92 (dd, 1H, *J* = 4.4, 3.6 Hz, H4′), 3.78 (m, 6H, DMTr), 3.73–3.67 (m, 1H, H5′), 2.37–2.22 (m, 4H, H2′, H6′), 1.96 (t, 1H, *J* = 2.4 Hz, H8′) 1.81 (d, 3H, *J* = 1.0 Hz, Me7). ^13^C-NMR (75 MHz, CDCl_3_), δ_ppm_: 164.2; 158.9; 150.7; 145.4; 135.9; 135.7; 130.4; 130.3; 127.9; 127.3; 113.3; 111.4; 87.6; 87.0; 84.2; 80.7; 72.0; 71.4; 71.0; 55.3; 40.7; 21.7; 12.4. MS-CI (*m*/*z*): 582.1 [M + H]^+^.

*5′-*O*-4,4′-Dimethoxytrityl-5′-C-(S)-propargyl-thymidine phosphoramidite* **9**. To a solution of 5′-(*S*)-*O*-DMTr-propargyl-thymidine **8** (990 mg, 1.69 mmol) in freshly distilled THF (12 mL) was added *N*-*N*-diisopropylethylamine (0.88 g, 6.8 mmol, 4 eq.) and (2-cyanoethyl)-*N*,*N*-diisopropylchlorophosphoramidite (0.81 g, 3.34 mmol, 2 eq.). The mixture was stirred for 45 min at room temperature under an argon atmosphere after which the precipitate was filtered off and the filtrate was diluted with ethyl acetate degassed with argon (40 mL). The organic phase was washed with an ice cold aqueous solution of Na_2_CO_3_ (10%, 10 mL), dried on MgSO_4_ and concentrated under reduced pressure. The crude product was purified by column chromatography (1:1 CH_2_Cl_2_/ethyl acetate, 1% Et_3_N) to yield **9** as a white foam (0.94 g, 70% yield). ^1^H-NMR (300 MHz, C_6_D_6_), δ_ppm_: 7.65 (br s, 1H, NH), 7.95–7.54 (m, 2H, DMTr), 7.46–7.39 (m, 4H, DMTr, H6), 7.12–7.00 (m, 4H, DMTr), 6.77–6.72 (m, 4H, DMTr), 6.55 (dd, 1H, *J* = 7.6, 6.0 Hz), 4.85–4.72 (m, 1H) 4.60–4.59 (dd, 1H, *J* = 3.3, 2.5 Hz), 3.74–3.88 (m, 1H), 3.50–3.41 (m, 2H), 3.31 (m, 6H, DMTr), 3.28–3.17 (m, 2H), 2.64–2.33 (m, 4H), 1.77 (m, 4H), 1.10–1.00 (m, 12H, *i*Pr). ^31^P-NMR (81 MHz, C_6_D_6_), δ_ppm_: 149.7, 148.8. MS-CI (*m*/*z*): 783.1 [M + H]^+^.

### 3.2. General ODN Synthesis and UV Thermal Denaturation Study

#### 3.2.1. Automated Synthesis

The oligonucleotides were assembled on CPG support (0.2 or 1 mmol scale) on a ABI 394-8 using the standard phosphoramidite chemistry and standard ABI program. After complete assembly of the oligonucleotide chain, cleavage and protective group removal were achieved with NH_4_OH (33%) at 25 °C for 24 h. The crude product was analyzed and purified by reversed phase HPLC on Waters X bridge OST C18 column (2.5 µm, 4.6 mm × 50 mm for analysis or 10 mm × 50 mm for purification scale) on a Waters apparatus (600 E pump system controller and a 996 photodiode array detector), using a gradient from 95% of A to 85% of A in B (A: TEAA buffer 0.05 M, pH 7.0; B: CH_3_CN). Analyses of the oligonucleotides were performed by mass spectrometry in MALDI TOF mode on a Waters Micromass MX spectrometer with THAP, 10% ammonium citrate as matrix.

#### 3.2.2. CuACC Protocol and Deprotection of 5′-C-Functionalized ODNs

All the CuAAC reactions were performed by using the following protocol unless stated otherwise. To an in-solution of **^alk^S_i_** (250 nmol in water) were added an azide derivative (degassed stock solution in MeOH, 3 equiv./alkyne), freshly prepared degassed aqueous solutions of CuSO_4_ (0.02 M, 0.4 equiv./alkyne) and sodium ascorbate (0.05 M, 2 equiv./alkyne), water and triethylammonium acetate buffer 0.1 M in a 4:1 ratio (up to 250 µL) and MeOH (up to 250 µL) to obtain a final volume of 500 µL. The tube containing the resulting mixture was placed in a block heater at 60 °C for the specified amount of time. The resulting ODNs solutions were desalted on Sephadex G-25 columns, concentrated and purified by reversed-phase preparative HPLC on a Waters X bridge OST C18 column (2.5 µm, 10 mm × 50 mm) using at a flow rate of 6 mL·min^−1^ using a gradient from 5% to 15% of acetonitrile in TEAA prior to deprotection or use into 3WJs assembly.

The introduction of **1** on **^alk^S_1_** was performed using Cu-TBTA complex (10 μL of a 10 mM solution in 55% aq. DMSO).

Pure compounds **^Asp^S_i_** were treated with a LiOH solution (0.5 mM, 500 μL) at r.t. for 15 h to remove the methyl ester, filtered on Sephadex G-25 columns and concentrated. Deprotection of pivaloyl group present on **^Ser^S_2_** after CuAAC was realized by addition of AMA (concentrated ammonia/40% aq. methylamine 1:1, *v*/*v*). The resulting solution was heated at 60 °C for 30 min and concentrated. All ODNs were finally purified again by reversed-phase preparative HPLC, evaporated and dissolved in water for subsequent analyses (see Table 2 for MALDI-TOF spectra see Figures S9–S15 in Supplementary Materials).

#### 3.2.3. PAGE Protocol

ODNs (8 nmol of each strand) were dissolved in the appropriate amount of phosphate buffer (10 mM Na_2_HPO_4_, 100 mM NaCl, 1 mM EDTA, pH = 7) and heated to 90 °C for 10 min before being allowed to cool down slowly to r.t. PAGEs were carried out on a 20% polyacrylamide gel (acrylamide/bisacrylamide 29:1) using TBE 1X as an electrophoresis buffer. Two gels were casted to run all the samples simultaneously in the same electrophoresis tank. ODNs were loaded with xylene cyanol and run for 3 h at 4 °C until the dye had migrated one third down the gel. Bands were revealed by UV-shadowing using a UV lamp (254 nm) and a fluorescent TLC plate and photographed.

#### 3.2.4. UV Thermal Denaturation Study

The thermal denaturation curves were recorded on a Varian CARY-300 Bio UV-vis spectrophotometer with the Cary Win UV thermal software (Agilent Technologies, Santa Clara, CA, USA). All measurements were performed in 1.0 cm path-length micro cuvettes in 10 mM Na_2_HPO_4_, 100 mM NaCl, 1 mM EDTA, pH = 7, buffer. The concentrations of the ODNs were calculated by measuring the absorbance at 260 nm and at 80 °C assuming unchanged molecular extinction coefficient for modified nucleotides. Samples were heated at 90 °C and cooled to 10 °C at a rate of 0.5 °C/min and then warmed up to 90 °C at the same rate.

## 4. Conclusions

We showed that incorporation of the convertible nucleotide 5′-C(*S*)-propargyl thymidine within DNA strands allowed the efficient installation of amino acids chains-like functionalities through a CuACC process. Hydroxyl (Ser), carboxyl (Asp) and imidazole (His) groups, potential mimics of the catalytic triad of protease, have been put together in a three way junction core. Thermal denaturation studies showed that the DNA structure was not affected by the functionalization and suggest that the decorated core of the junction could be large enough to ensure a catalytic activity. As a correct orientation of the pendent functionalities is crucial to achieve efficient and cooperative catalysis, molecular modeling studies are currently performed to further determine the optimum positioning of the amino acids residues. Furthermore, DNA 3WJ are interesting tiles since they can be used alone or in a 2D network when connected with sticky ends and even as 3D structure when assembled in DNA tetrahedron. Evaluation of amide bond hydrolysis by these systems is underway.

## Figures and Tables

**Table 1:** Sequences and melting temperatures of 5′-C-functionalized duplexes and three way junctions (3WJs).

Sequences	*T*_m_ °C	*T*_m_ °C (Δ*T*_m_ °C) ^2^			
	(Δ*T*_m_ °C) ^1^ Duplex	3WJ			
**^wt^S_1_**/**^wt^S′_1_** ^5′^GCGACCTATTGCAAGTGG ^3′^CGCTGGATAACGTTCACC	66 (*T*_m_1)	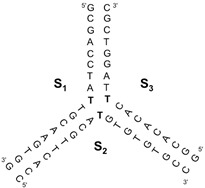			**^wt^S_1_**, **^wt^S_2_**, **^wt^S_3_** (**^wt^3WJ**) 43 (*T*_m_4)
**^wt^S_2_**/**^wt^S′_2_** ^5′^CCACTTGCATGTGTGTGCC ^3′^GGTGAACGTACACACACGG	69 (*T*_m_2)				
**^wt^S_3_**/**^wt^S′_3_** ^5′^GGCACACACTTAGGTCGC ^3′^CCGTGTGTGAATCCAGCG	68 (*T*_m_3)				
**^alk^S_1_**/**^wt^S′_1_** ^5′^GCGACCTATTGCAAGTGG ^3′^CGCTGGATAACGTTCACC	65 (−1)	**^alk^S_1_**, **^wt^S_2_**, **^wt^S_3_** 43 (0) **^wt^S_1_**, **^alk^S_2_**, **^alk^S_3_** 43 (0)	**^wt^S_1_**, **^alk^S_2_**, **^wt^S_3_** 43 (0) **^alk^S_1_**, **^wt^S_2_**, **^alk^S_3_** 42 (−1)	**^wt^S_1_**, **^wt^S_2_**, **^alk^S_3_** 43 (0) **^alk^S_1_**, **^alk^S_2_** **^wt^S_3_** 43 (0)	**^alk^S_1_**, **^alk^S_2_**, **^alk^S_3_** (**^alk^3WJ**) 43 (0)
**^alk^S_2_**/**^wt^S′_2_** ^5′^CCACTTGCATGTGTGTGCC ^3′^GGTGAACGTACACACACGG	68 (−1)				
**^alk^S_3_**/**^wt^S′_3_** ^5′^GGCACACACTTAGGTCGC ^3′^CCGTGTGTGAATCCAGCG	66 (−2)				
**^Asp^S_1_**/**^wt^S′_1_** ^5′^GCGACCTAT^Asp^TGCAAGTGG ^3′^CGCTGGATA ACGTTCACC	64 (−2)	**^Asp^S_1_**, **^wt^S_2_**, **^wt^S_3_** 42 (−1) **^Asp^S_1_**, **^Ser^S_2_**, **^wt^S_3_** 43 (0)	**^wt^S_1_**, **^Ser^S_2_**, **^wt^S_3_** 44 (+1) **^Asp^S_1_**, **^wt^S_2_**, **^His^S_3_** 43 (0)	**^wt^S_1_**, **^wt^S_2_**, **^His^S_3_** 44 (+1) **^wt^S_1_**, **^Ser^S_2_**, **^His^S_3_** 44 (+1)	**^Asp^S_1_**, **^Ser^S_2_**, **^His^S_3_** 43 (0)
**^Ser^S_2_**/**^wt^S′_2_** ^5′^CCGCTTGCAT^Ser^GTGTGTGCC ^3′^GGCGAACGTACACACACGG	68 (−1)				
**^His^S_3_**/**^wt^S′_3_** ^5′^GGCACACACT^His^TAGGTCGC ^3′^CCGTGTGTGAATCCAGCG	68 (0)				

^1^ Values of Δ*T*_m_ are calculated relatively to *T*_m_1, *T*_m_2 and *T*_m_3 for duplex made with **^alk^S_1_**, **^alk^S_2_**, **^alk^S_3_**; and **^Asp^S_1_, ^Ser^S_2_** and **^His^S_3_** respectively; ^2^ Values of Δ*T*_m_ are calculated relatively to *T*_m_4 for all 3WJs.

**Table 2:** CuAAc conditions reactions, HPLC and MALDI-TOF characterization of wt and 5′-C-functionalized ODNs.

Sequence		CuAAC Conditions	t_r_ (min)	[M − H]^−^ Calcd.	[M − H]^−^ Found
**^wt^S_1_**	^5′^GCGACCTATTGCAAGTGG	-	6.35 ^1^	5538.6	5539.9
**^wt^S_2_**	^5′^CCACTTGCATGTGTGTGCC	-	6.40 ^1^	5769.7	5771.0
**^wt^S_3_**	^5′^GGCACACACTTAGGTCGC	-	6.27 ^1^	5483.6	5484.3
**^alk^S_1_**	^5′^GCGACCTA**T**TGCAAGTGG	-	6.52 ^2^	5576.6	5577.0
**^alk^S_2_**	^5′^CCACTTGCATG**T**GTGTGCC	-	6.57 ^2^	5807.8	5806.7
**^alk^S_3_**	^5′^GGCACACAC**T**TAGGTCGC	-	6.49 ^2^	5521.6	5520.8
**^Asp^S_1_**	^5′^GCGACCTA**T^Asp^**TGC AAGTGG	**1**, TBTA, 1 h	5.04 ^2^	5691.7	5692.3
**^Asp^S_2_**	^5′^CCACTTGCATG**T^Asp^**GTGTGCC	**1**, 3 h	5.18 ^2^	5922.9	5923.1
**^Asp^S_3_**	^5′^GGCACACAC**T^Asp^**TAGGTCGC	**1**, 3 h	4.75 ^2^	5636.7	5636.8
**^Ser^S_2_**	^5′^CCACTTGCATG**T^Ser^**GTGTGCC	**3**, 1.5 h	5.74 ^2^	5894.9	5893.7
**^His^S_3_**	^5′^GGCACACAC**T^His^**TAGGTCGC	**2**, 0.5 h	6.02 ^2^	5658.7	5660.4

^1^ Analyses were performed on a Waters 717 plus autosampler and UV 600 controller at a flow rate of 1 mL·min^−1^ using a gradient of acetonitrile from 5% to 15% in 0.05 M aqueous triethylammonium acetate (pH 7) for 15 min; ^2^ Analyses were performed on an Alliance Waters 2695 Separation module (Waters, Milford, MA, USA) at a flow rate of 1 mL·min^−1^ using a gradient of acetonitrile from 5% to 15% in 0.05 M aqueous triethylammonium acetate (pH 7) for 15 min.

## Supplementary Materials

The following are available online at http://www.mdpi.com/1420-3049/21/9/1082/s1.
