# Supplementary material for: DNA Three Way Junction Core Decorated with Amino Acids-Like Residues-Synthesis and Characterization
Source: Molecules. 2016 Aug 23;21(9):1082. doi: 10.3390/molecules21091082 (PMC6274049; doi:10.3390/molecules21091082)
Supplement: Supplementary file 1 [file molecules-21-01082-s001.pdf]

Supplementary Materials: DNA Three Way Junction Core Decorated with Amino Acids-Like Residues-Synthesis and Characterization

Claudia Addamiano, Béatrice Gerland, Corinne Payrastre and Jean-Marc Escudier

1. HPLC Chromatograms of ODN $^{alk}S_1$, $^{alk}S_2$, $^{alk}S_3$, $^{alk}S_1$, protected $^{Asp}S_1$, $^{Asp}S_1$, protected $^{Ser}S_2$, $^{Ser}S_2$ and $^{His}S_3$

Analyses were performed on an Alliance Waters 2695 Separation moduler at a flow rate of 1 mL min^{-1} usinGa gradient of acetonitrile from 5% to 15% in 0.05 M aqueous triethylammonium acetate (pH 7) for 15 min.

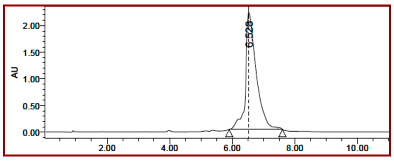

Figure S1. $^{alk}S_1$ 5′-GCGACCTATTGCAAGTGG-3′.

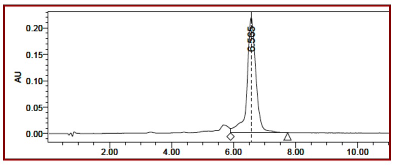

Figure S2. $^{alk}S_2$ 5′-CCACTTGCATGTGTGTGCC-3′.

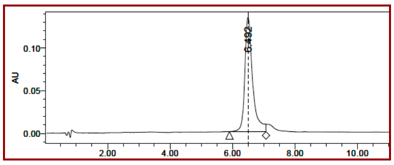

Figure S3. $^{alk}S_3$ 5′-GGCACACACTTAGGTCGC-3′.

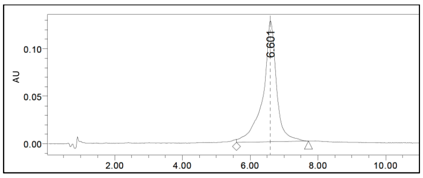

Figure S4. $^{Asp}S_1$ methyl ester protected 5′-GCGACCTAT^{SerOMe}TGCAAGTGG-3′.

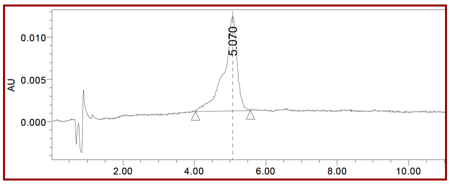

Figure S5. $^{Asp}S_1$ 5′-GCGACCTAT^{Ser}TGCAAGTGG-3′.

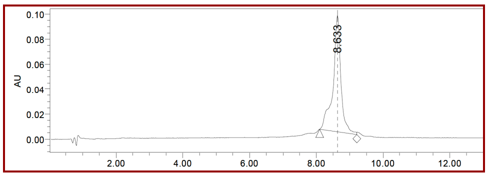

Figure S6. $^{Ser}S_2$ Piv protected 5′-CCACTTGCATGT^{SerPiv}GTGTGCC-3′.

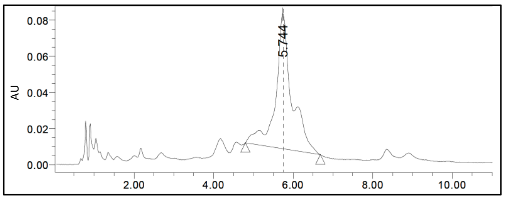

Figure S7. $^{Ser}S_2$ 5′-CCACTTGCATGT^{Ser}GTGTGCC-3′.

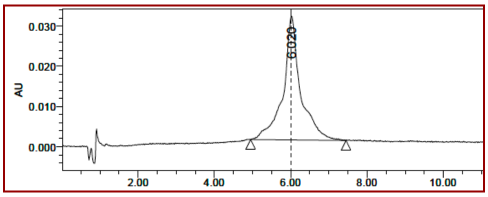

Figure S8. $^{His}S_3$ 5′-GGCACACACT^{His}TAGGTCGC-3′.

2. MALDI-TOF Spectra of ODN $^{alk}S_1$, $^{alk}S_2$, $^{alk}S_3$, $^{alk}S_1$, $^{Asp}S_1$, protected $^{Ser}S_2$, $^{Ser}S_2$ and $^{His}S_3$.

Analyses of the oligonucleotides were performed by mass spectrometry in MALDI TOF mode on a Waters Micromass MX spectrometer with THAP, 10% ammonium citrate as matrix.

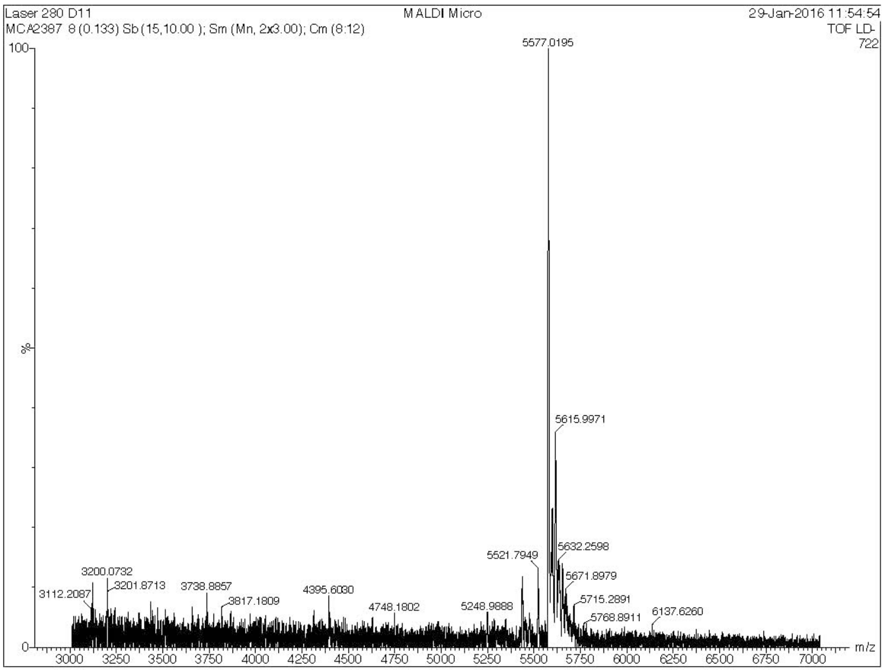

Figure S9. $^{alk}S_1$ 5′-GCGACCTA<u>T</u>TGCAAGTGG-3′.

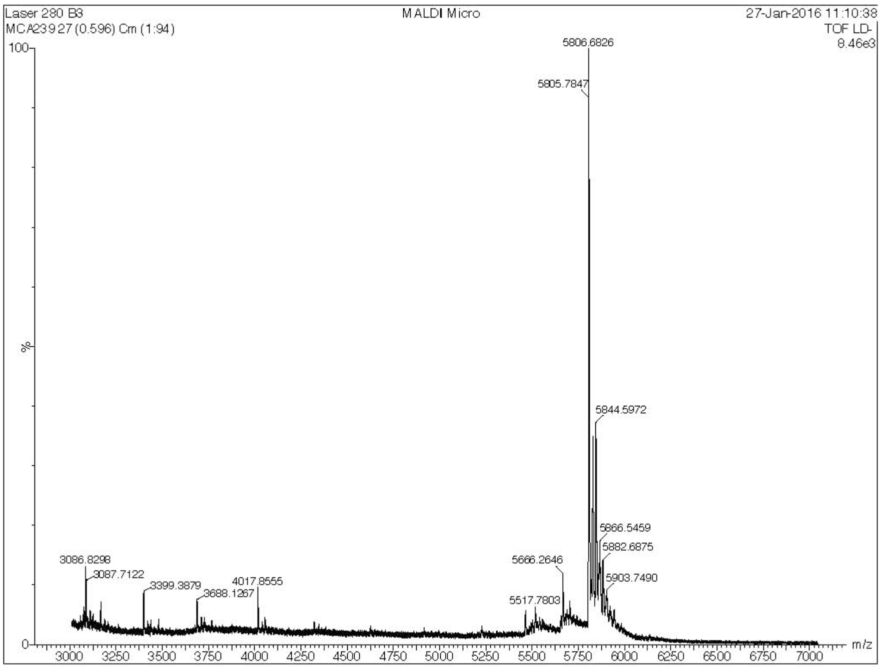

Figure S10. $^{alk}S_2$ 5′-CCACTTGCATG**TG**TGTGCC-3′.

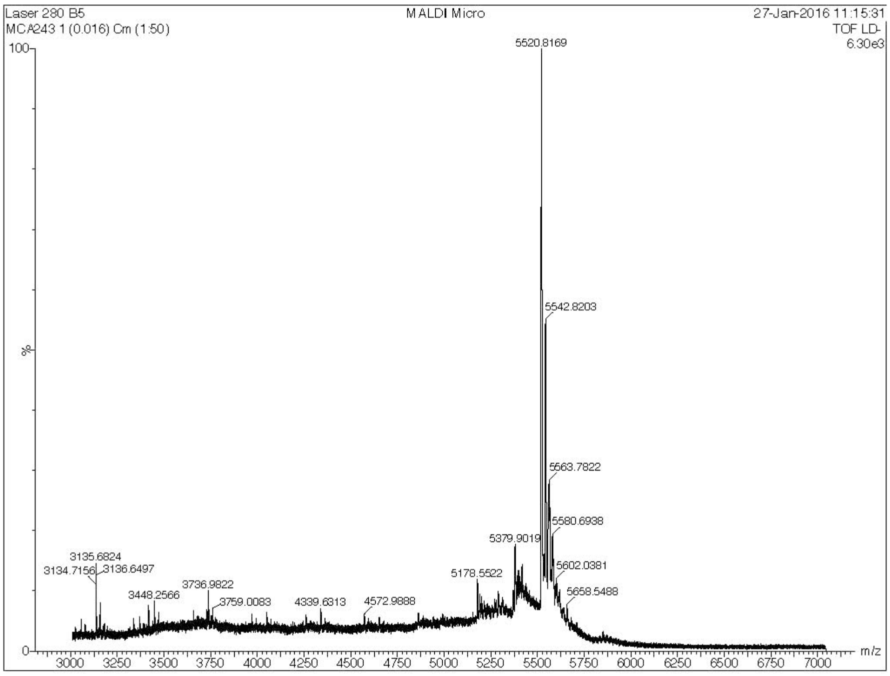

Figure S11. $^{alk}S_3$ 5′-GGCACACACTTAGGTCGC-3′.

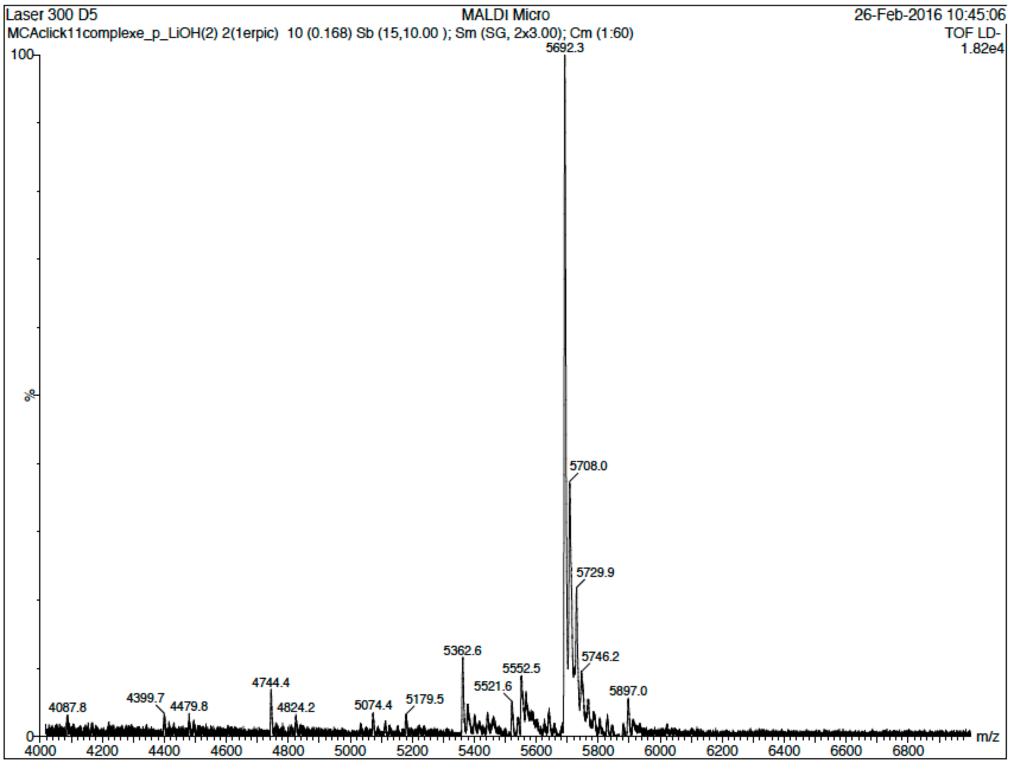

Figure S12. $^{Asp}S_1$ 5′-GCGACCTAT^{Asp}TGCAAGTGG-3′.

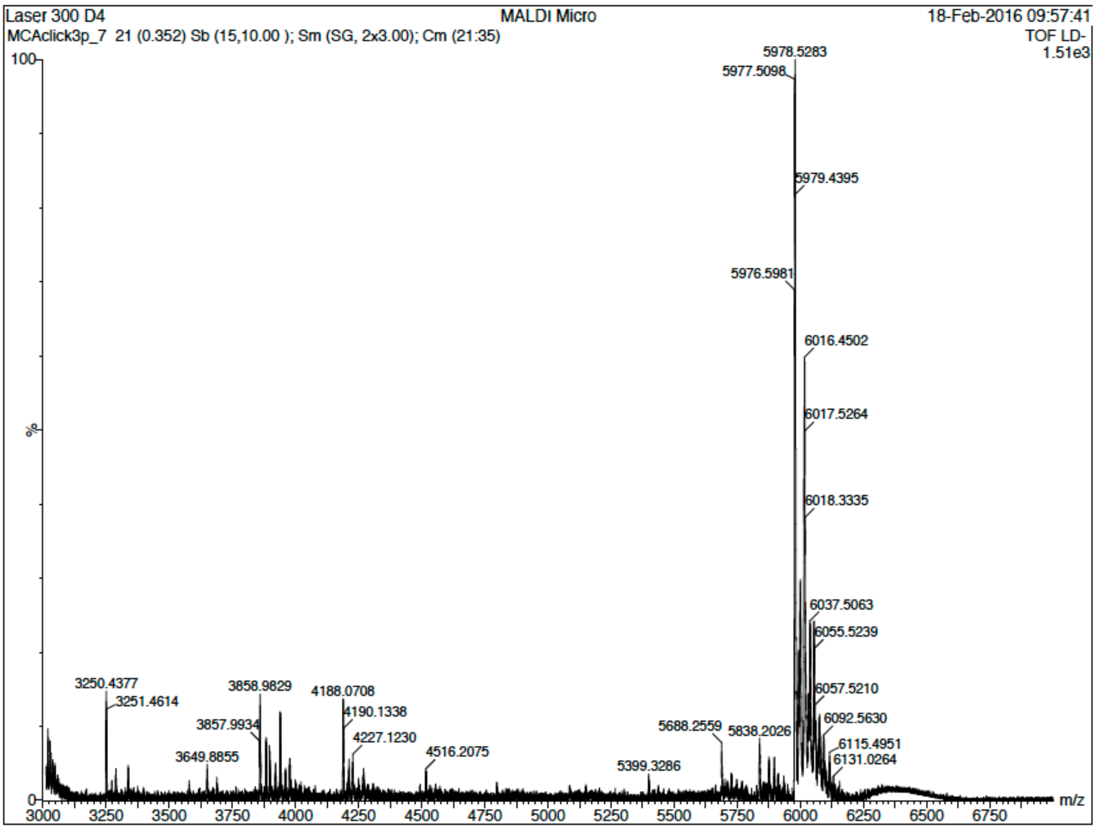

Figure S13. $^{Ser}S_2$ Piv protected 5′-CCACTTGCATGT^{SerPiv} GTGTGCC-3′.

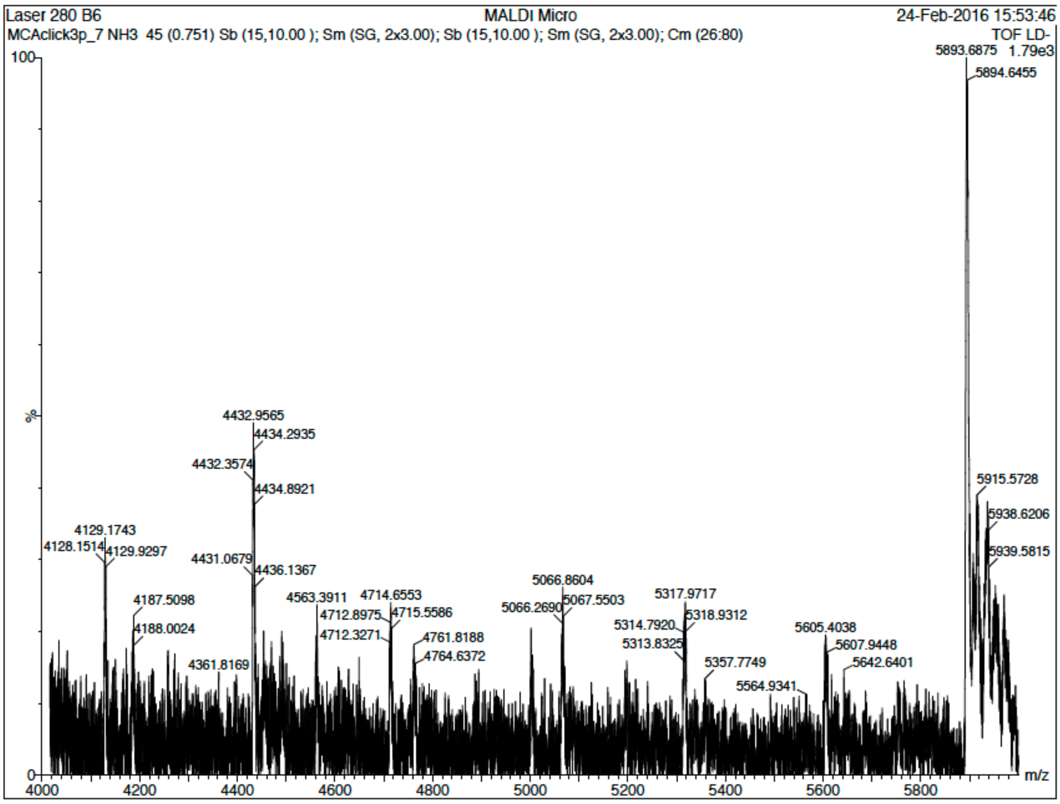

Figure S14. $^{Ser}S_2$ 5′-CCACTTGCATGT^{Ser} GTGTGCC-3′.

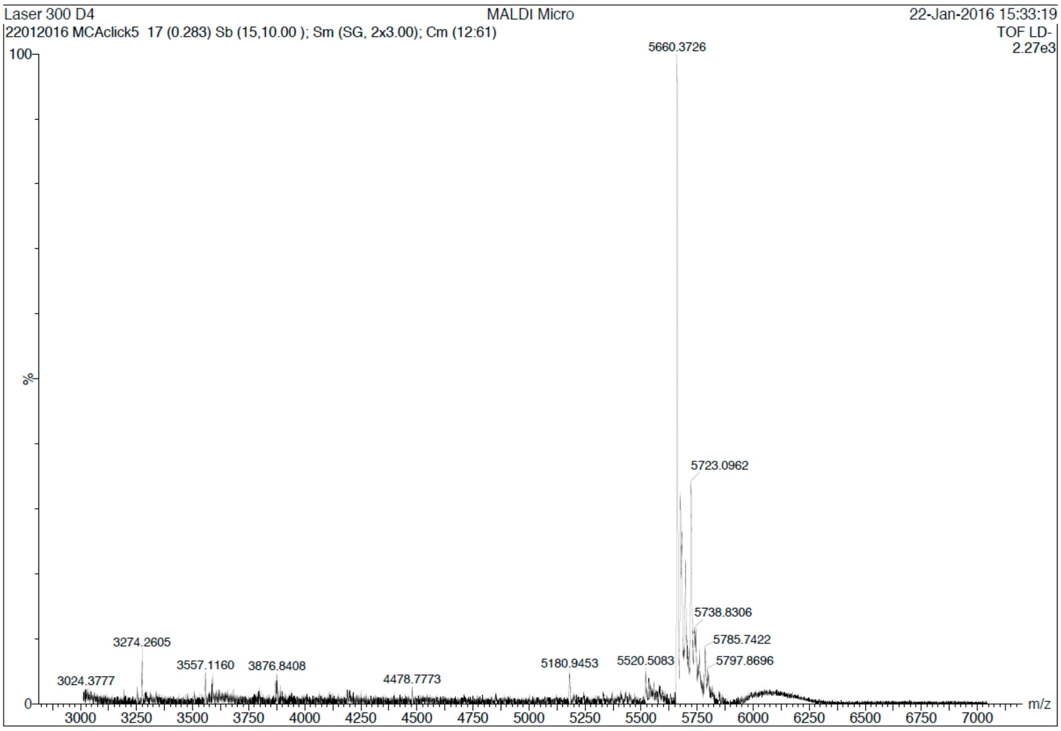

Figure S15. $^{His}S_3$ 5′-GGCACACACT^{His}TAGGTCGC-3′.
